# Effects of dandelion (Taraxacum sp.,) supplements on lactation performance, antioxidative activity, and plasma metabolome in primiparous dairy cows

**DOI:** 10.5713/ab.22.0061

**Published:** 2022-09-02

**Authors:** Yan Li, Jie Mei, Jiaqi Wang, Hongyun Liu

**Affiliations:** 1 College of Animal Sciences, Zhejiang University, Hangzhou 310058, China

**Keywords:** Dairy Cows, Dandelion, Lactation Performance, Metabolomics, Plasma

## Abstract

**Objective:**

This study evaluated the effects of dandelion supplements on lactation performance, circulating antioxidative activity and plasma metabolomics in primiparous dairy cows.

**Methods:**

A total of 60 mid-lactation dairy cows (milk yield = 34.29±0.34 kg/d; days in milk = 151.72±2.36 days) were divided into 4 treatment groups randomly, comprising the addition of dandelion at 0, 100, 200, 400 g/d per head. The experiment lasted for 8 weeks with an extra 10 days’ pre-feeding period. Milk and blood samples were collected, and plasma samples were selected to perform metabolomics analysis.

**Results:**

Supplementing 200 g/d of dandelion increased the yield of milk and lactose (p≤ 0.05). The milk somatic cell counts (p≤0.05) were lower in all dandelion groups than those in the control group. The activity of glutathione peroxidase (p≤0.05) and superoxide dismutase (p≤0.05) were increased and plasma malondialdehyde (p = 0.01) was decreased when cows were fed 200 g/d dandelion. Plasma metabolomics analysis showed that 23 hub differential metabolites were identified in the 200 g/d dandelion group. These metabolites such as ribose, glutamic acid, valine, and phenylalanine were enriched in D-glutamine and D-glutamate metabolism (p = 0.06, impact value = 1), phenylalanine, tyrosine, and tryptophan biosynthesis (p = 0.05, impact value = 0.5), and starch and sucrose metabolism (p = 0.21, impact value = 0.13). Moreover, correlation analysis showed that circulating ribose, mannose, and glutamic acid were positively related to milk yield.

**Conclusion:**

Dandelion supplementation could improve lactation performance and elevate the plasma carbohydrate and amino acids metabolism and antioxidative activity. Supplementation of 200 g/d dandelion is recommended for lactating dairy cows.

## INTRODUCTION

Dandelion (*Taraxacum mongolicum* Hand.-Mazz.), a member of the Asteraceae family, contains various biological phytochemicals that are beneficial to animal health [1]. Generally, it is commonly used as a medicinal herb owing to its diuretic, antibacterial, anti-inflammatory, antidepressant, antiallergy, antioxidant, and choleretic properties. Previous studies have associated these properties with the presence of an array of secondary metabolites, including sesquiterpene lactones, phenolic compounds, polysaccharides, and flavonoids [2–4]. The use of medicinal herbs, as well as their associated secondary metabolites, in the livestock sector has attracted interest from animal researchers and practitioners [5], due to the existing challenges encountered during the application of these products in animal production [6].

Several studies have described the use of medicinal herbs in animal husbandry. The milk yield, rumen fermentation, and immune function were improved when dairy cows were fed with medicinal plants like triterpene saponins and *Perilla frutescens* leaf [7,8]. The addition of dandelion to the diet of weaning pigs was found to improve the growth rate, the efficiency of feed intake and energy digestibility [9]. Besides, dandelion extracts could enhance intestinal digestion and absorption in golden pompano [10]. However, the underlying mechanisms have not been explored sufficiently in these researches.

Metabolomics is a useful method to explore the essential and complex changes in various biological systems. In our previous study, the rumen fluid metabolome was found to improve the tryptophan metabolism, starch and sucrose metabolism, and pentose phosphate pathway [11]. However, information about the effects of dandelion on lactation parameters and plasma metabolomics in dairy cows is relatively limited. Hence, we sought to evaluate the effect of dandelion supplementation on lactation performance, plasma variables and metabolome in dairy cows.

## MATERIALS AND METHODS

### Animal care

The experimental procedures were approved by the Animal Care Committee of Zhejiang University and complied with the guidelines of animal research (Protocol No. ZJU20160379, Hangzhou, China).

### Animals and experimental design

A total of 60 mid-lactating primiparous dairy cows were selected and blocked based on milk yield and days in milk (milk yield = 34.29±0.34 kg/d; days in milk = 151.72±2.36 days), then randomly assigned to 4 dietary groups (n = 15/treatment). The cows were maintained on the same basal diet supplemented with 0, 100, 200, or 400 g/d of dandelion per head. We supplemented the dandelion individually before the total mixed ration (TMR) was fed and confirmed that the dairy cows had eaten all the dandelion. The dandelion plants, comprising whole-crop in an air-dried form, including stalks, leaves, and flowers, were planted, and harvested by the dairy farm of Yinxiang Weiye Group. Dandelion was ground, sieved through a 1-cm screen, then stored in sealed bags until feeding. The nutrient composition of basal TMR and the bioactive compounds of dandelion are outlined in supplemental Table 1 and supplemental Table 2. Cows were housed in a sand-bedded free-stall barn, with feed available *ad libitum* and free access to water. Milking and feeding procedures were performed 3 times a day (at 07:00, 14:30, and 21:30), with an 8 week formal feeding and a 10 days’ pre-feeding period.

### Sampling and analysis

Dry matter intake was calculated daily by the TMR and refusals of each group. During the trial, TMR samples were collected on the second and third day of each week, then mixed to make a composite sample for each week and treatment. Samples of individual concentrates, as well as forages, were also collected weekly and immediately stored at −20°C. Part of the samples were dried in a forced-air oven, at 65°C for 48 h, then used for the determination of dry matter (DM). Others were ground in a Wiley Mill (1-mm screen; Thomas Scientific, Swedesboro, NJ, USA) and used for compositional analyses. Composite samples of forages and concentrates were analyzed for DM, crude protein, and ether extract [12]. The content of acid detergent fiber and neutral detergent fiber were determined by an Ankom A200 apparatus (Ankom Technology, Macedon, NY, USA) with heat-stable amylase and sodium sulfite (Fisher Scientific, Waltham, MA, USA) and expressed inclusive of residual ash [13].

Milk samples were collected on the sixth day of each week, 50 mL of milk sample was collected from each cow, and samples across the 3 milking times per day at a ratio of 4:3:3 as earlier described [14]. The 50 mL composite milk samples were stored in a sample bottle, with the addition of 0.6 mg/mL potassium dichromate to act as a preservative, then stored at 4°C to await further analysis. Thereafter, an automatic ultrasonic milk composition analyzer (Bentley Instruments, Chaska, MN, USA) was used to determine milk components, including protein, fat, lactose contents, as well as somatic cell count (SCC), and milk urea nitrogen.

Blood samples (10 mL) were collected from the coccygeal vein into evacuated tubes containing sodium heparin before morning feeding on the fifth day of week 1, 4, and 8. Plasma was then obtained from the sampled blood, by centrifugation at 3,000×*g* for 10 min at 4°C and stored at −20°C for further analysis. Glucose, nonesterified fatty acids, blood urea nitrogen, total protein, bilirubin, albumin, triglyceride, globulin, β-hydroxybutyrate, creatinine, aspartate aminotransferase, alanine aminotransferase, and alkaline phosphatase concentrations in plasma samples were analyzed by an auto-analyzer 7020 (Hitachi High-Technologies Corp., Tokyo, Japan) according to the method described in previous study [15]. Commercial assay kits of Jiangsu Nanjing Biological Technology Co. Ltd., Jiangsu, China, were then used to determine the enzymatic activity of malondialdehyde (MDA), superoxide dismutase (SOD), glutathione peroxidase (GSH-Px), and total antioxidant capacity as previously described [16].

### Metabolomics analysis

Sixteen plasma samples (8 in control group, 8 in 200 g/d dandelion group) were selected to perform the metabolomics analysis according to the phenotype of milk yield, feed intake, and milk quality. Fifty μL sample was transferred into a 1.5 mL tube, then added 200 μL of pre-chilled methanol containing 5 μL internal standard (adenosine, 0.5 mg/mL stock solution) and vortexed for 30 s. Samples were sonicated in ice water for 10 minutes. After centrifugation at 4°C and 10,000 rpm for 15 minutes, 180 μL of the supernatant was transferred to a fresh tube. To prepare quality control (QC) sample, 30 μL of each sample was taken out and combined. After evaporation in a vacuum concentrator, 30 μL of Methoxyamination hydrochloride (20 mg/mL in pyridine) was added and then incubated at 80°C for 30 minutes, then derivatized by 40 μL Bis(trimethylsilyl)trifluoroacetamide (BSTFA) reagent (1% Trimethylchlorosilane, v/v) for 1.5 hours at 70°C. Gradually cooling samples to room temperature and added 5 μL fatty acid methyl esters (in chloroform) to the QC sample. All samples were analyzed by gas chromatography combined with a time-of-flight mass spectrometer (GC-TOF-MS).

GC-TOF-MS analysis was performed using an Agilent 7890 gas chromatograph coupled with a time-of-flight mass spectrometer. The system utilized a DB-5MS capillary column. One μL aliquot sample was injected in a splitless manner. Helium was used as the carrier gas, the front inlet purge flow was 3 mL/min, and the gas flow rate through the column was 1 mL/min. The initial temperature was kept at 50°C for 1 minute, then increased to 310°C at a rate of 20°C/min, held at 310°C for 8 minutes. The temperature of the injection line, transfer line, and ion source was 280°C, 280°C, and 250°C, respectively. In the electron impact mode, the energy was −70 eV. After a solvent delay of 4.8 minutes, mass spectrometry data were acquired in the full scan mode with the range of 50 to 500 m/z.

Raw data analysis, including peak extraction, baseline ad justment, deconvolution, comparison, and integration, was finished with Chroma TOF (V 4.3×, LECO) software and the LECO-Fiehn Rtx5 database was used for metabolite identification by matching the mass spectrum and retention index. Finally, less than half of the peaks detected in QC samples or relative standard deviation >30% in QC samples was removed.

### Statistical analysis

All data were analyzed by the mixed model (SAS 9.2, 2000) and checked for normality by the UNIVARIATE procedure. A randomized block design, with repeated measures, was used for the analyses, with the treatment and week as the fixed effects, the cow as a random effect. Contrasts were constructed to examine treatment effects and dandelion supplementation levels, with orthogonal polynomials accounting for the unequal spacing of dandelion levels. The differences between the groups were evaluated by Tukey’s multiple range test. Data are expressed as least squares means and standard errors of the mean. Correlation analyses between lactation parameters and plasma metabolites were calculated using spearman’s correlation test. All analyses were performed at p≤0.05 for determination of statistical significance, with 0.05<p≤0.10 indicated trends.

## RESULTS

### Lactation performance and feed intake

**Figure 1:**
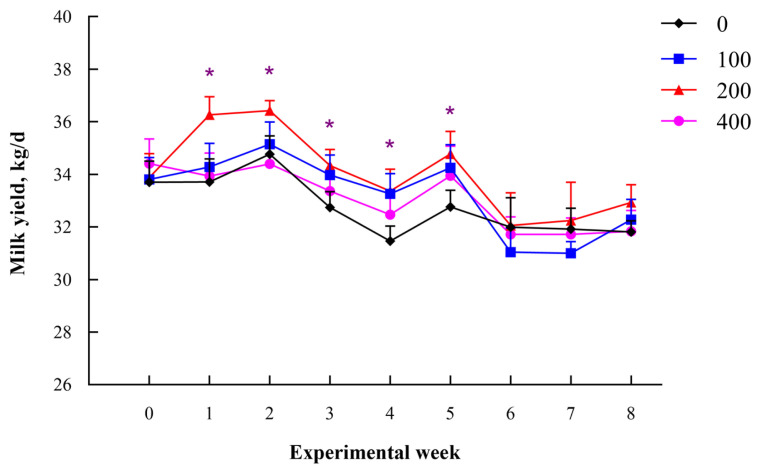
Changes in milk yield of mid-lactating cows fed on a basal diet with 0, 100, 200, 400 g/d of dandelion supplemented. Bars indicate standard error of the mean; Asterisk (*) indicates significant differences (p<0.05) between the control (0) and dandelion (200 g/d) groups.

A quadratic increase in milk yield (p = 0.04) was observed, with the highest level recorded in the group fed on 200 g/d dandelion (Table 1). In addition, the energy corrected milk (ECM) (p = 0.03) and lactose (p = 0.05) yield were higher in cows fed on 200 g/d dandelion relative to those in the control group. The SCC (p = 0.01) was lower in all dandelion addition groups. The yield of milk was higher in the whole experiment period, while the results showed significant in the first 5 weeks when cows were fed on 200 g/d of dandelion (Figure 1).

### Plasma variables and antioxidative parameters

The concentration of glucose was higher in the 200 and 400 g/d groups (p = 0.05), compared with the 0 and 100 g/d groups (Table 2). Plasma SOD activity was increased, and MDA concentration was decreased in the group supplemented with 400 g/d dandelion (p = 0.05). Moreover, dietary 200 g/d dandelion supplementation significantly increased plasma activity of GSH-Px (p = 0.05), and SOD (p = 0.05), decreased the concentration of MDA (p = 0.01).

### GC/MS analysis of the plasma

**Figure 2:**
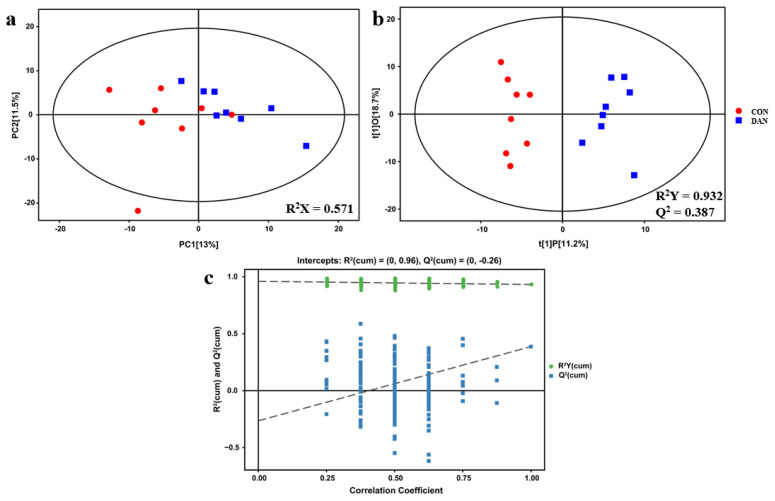
The multivariate statistical analysis of CON and DAN plasma metabolome. The red circle represents DAN, while the blue box represents CON. This figure contains the PCA score map (a) OPLS-DA score plot (b) and permutation plot (c) derived from the metabolite profiles of dairy cows. PCA, principal component analysis; OPLS-DA, orthogonal partial least squares discriminant analysis. CON, control group; DAN, 200 g/d of dandelion supplemented group.

According to the results of milk performance in dairy cows, optimal supplementation dosage of dandelion was confirmed, and 16 plasma samples were selected from the 0 (CON) and 200 g/d (DAN) groups. The analysis of orthogonal partial least squares-discriminant analysis (OPLS-DA) and principal component analysis of the metabolic profiles showed a separate trend between the CON and DAN groups (Figure 2), suggesting that the model of plasma metabolomics could be used to identify the difference between the two groups.

### Plasma differential metabolites between CON and DAN groups

In general, 23 differential metabolites were identified in the CON and DAN groups (p<0.05, VIP>1), with 19 metabolites upgraded and 4 downgraded (Table 3), including amino acids (valine, cystine, glutamic acid, phenylalanine, and threonine), organic acids (palmitic acid, methylmalonic acid, and salicylic acid), and sugars (mannose, glucose, glucose-1-phosphate, and ribose).

### Characterization of metabolic pathways

**Figure 3:**
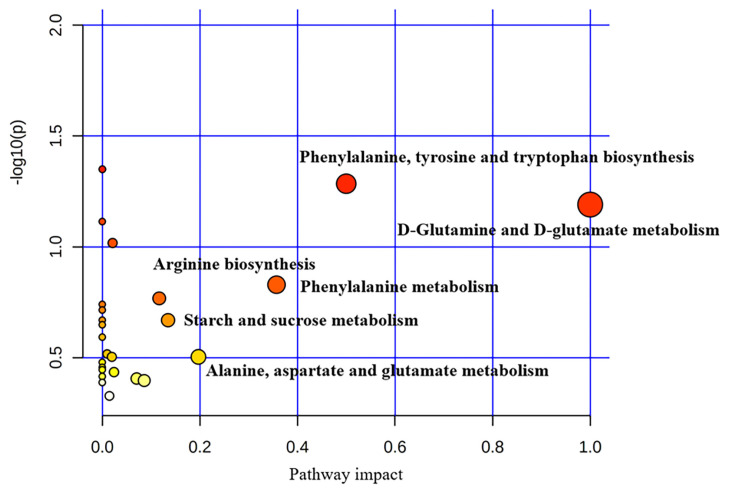
The metabolome view map of significant metabolic pathways characterized in plasma for cows in the CON and DAN group. The x-axis represents pathway enrichment, and the y-axis represents pathway impact. Larger sizes and higher impact values, respectively. CON, control group; DAN, 200 g/d of dandelion supplemented group.

The plasma metabolome view map of significant metabolic pathways characterized in the CON and DAN group is shown in Figure 3. Enrichment and pathway topology analysis showed that 6 pathways exhibited an important value at the meaningful level (impact value >0.10): Phenylalanine, tyrosine and tryptophan biosynthesis (p = 0.05, impact value = 0.5), D-glutamine and D-glutamate metabolism (p = 0.06, impact value = 1), Starch and sucrose metabolism (p = 0.21, impact value = 0.13), Phenylalanine metabolism (p = 0.15, impact value = 0.36), Alanine, aspartate and glutamate metabolism (p = 0.31, impact value = 0.20), Arginine biosynthesis (p = 0.17, impact value = 0.12).

### Correlation analysis between lactation performance and plasma metabolites

**Figure 4:**
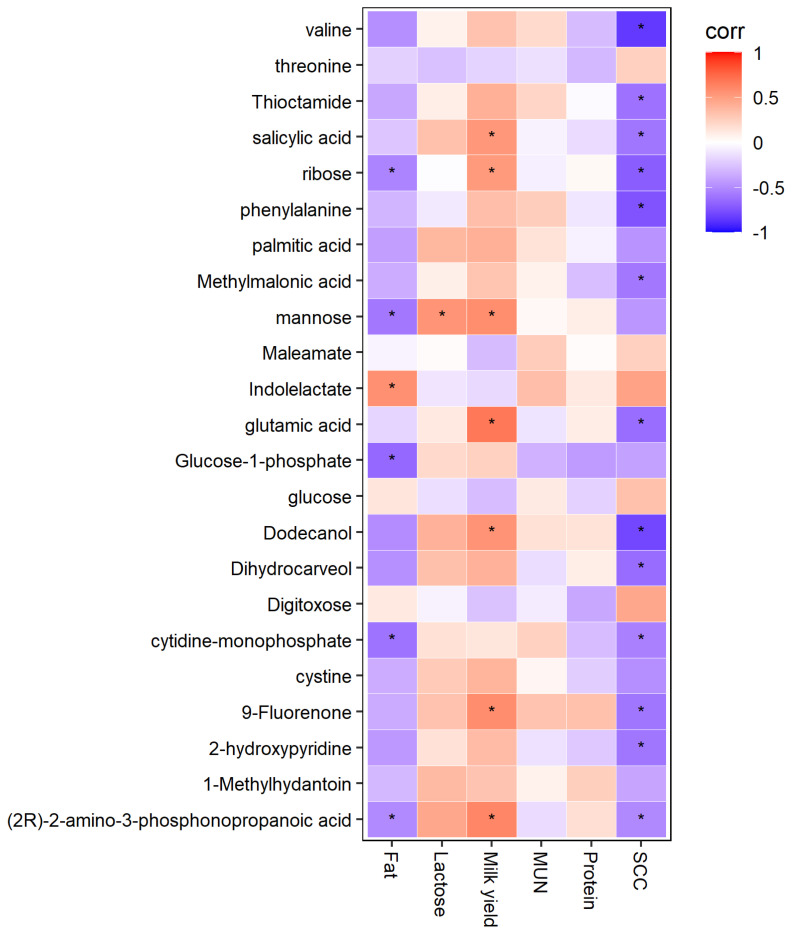
Correlation analyses between the lactation performance and plasma differential metabolites. The red squares represent the positive correlations while the blue ones represent the negative correlations. * p<0.05. MUN, milk urea nitrogen; SCC, somatic cell counts.

Correlation analyses between the lactation performance and plasma differential metabolites is shown in Figure 4. Ribose, mannose, and glutamic acid were positively correlated with milk yield (p<0.05). Mannose was positively correlated with lactose (p<0.05). Ribose, mannose, glucose-1-phosphate were negatively correlated with fat (p<0.05). Most of differential metabolites were negatively correlated with SCC (p<0.05).

## DISCUSSION

Lactation performance is an important and comprehensive parameter for dairy husbandry. In previous study, we have identified the bioactive compounds in dandelion, among them, the most abundant compound is β-D-glucopyranoside, which functioned to stimulate the process of digestion. Besides, the rumen microorganisms that correlated with the degrading of cellulose and structure carbohydrate were enriched when the dandelion was supplemented [11]. Another study has reported an increase in growth performance, the efficiency of feed intake, and energy digestibility in animals fed on dandelion [9]. Functional foods like dandelion can enhance digestibility, owing to the presence of bitter sesquiterpene lactones [17]. Hence, the increase in ECM and lactose yield in dandelion-fed dairy cows may be attributed to the higher milk yield and digestion-enhancing activities. These results indicated that the lactation performance could be improved with the supplementation of dandelion on mid-lactation dairy cows.

Notably, supplementing feed with dandelion led to a sig nificantly lower SCC in dairy cows in the treated group relative to controls, suggesting an important role of dandelion in improving milk quality. Dandelion flowers are a potential source of natural antioxidants due to the higher content of phenolic compounds [1]. The effects of the crude extract of flowers in inhibiting the damage caused by reactive oxygen species and the damage caused by nitric oxide were attributed to caffeic acid, luteolin 7-glucoside, flavonoid luteolin, and chlorogenic acid [18–19]. Moreover, dietary dandelion supplementation significantly increased plasma concentration of GSH-Px and SOD, decreased the MDA. It is generally accepted that SOD plays a significant role in the conversion of oxygen radicals to peroxides [20], whereas MDA is an indicator of oxidative stress owing to its activity as a lipid peroxidation product [21]. Previous studies have shown that blood MDA and SOD are negatively correlated [22]. In this study, the low MDA and high SOD concentrations in plasma obtained from cows in the dandelion group might indicate that this plant reduces oxidative stress in cows. Besides, 2-Hydroxypyridine was negatively correlated with SCC, which could combine with copper to function as the most potent SOD mimic [23]. Therefore, supplementing feed with dandelion might improve the antioxidant activities of dairy cows, which could then enhance the health status and lactating performance.

Metabolomics analysis revealed that the levels of valine, phenylalanine and glutamate acid were enriched in the DAN group, suggesting that an increasing trend of milk protein synthesis. A previous study has shown that feeding rumen-degradable valine could increase the milk yield in late-lactating dairy cows [24]. Besides, in MAC-T cells, valine and phenylalanine could stimulate milk protein synthetic and energy-mediated pathways differentially [25]. The results showed that phenylalanine and metabolism phenylalanine, tyrosine, and tryptophan biosynthesis were mainly affected by phenylalanine and tyrosine. Phenylalanine could also be able to convert into Tyr in the mammary gland of ruminant animals and regulate the synthesis or secretion of lipase, trypsin, and α-amylase through mRNA translation initiation factors ribosomal protein S6 kinase 1 (S6K1) and 4E-binding protein 1 (4EBP1) in dairy calves [26,27]. These findings indicated that the significantly different metabolites could play an important role in the milk synthesis.

For the metabolic pathways, phenylalanine, tyrosine and tryptophan biosynthesis, D-glutamine and D-glutamate, starch and sucrose metabolism were identified in this study. D-glutamine and D-glutamate metabolism were affected by glutamate, which could be transferred from glutamic acid. As an NEAA, previous studies had shown that glutamate could limit the synthesis of protein [28] and decrease the SCC of milk and improve the health status of high-yielding dairy cows [29]. Correlation analysis results also revealed that the ribose, mannose, and glutamic acid were positively correlated with milk yield. Thus, it is possible to infer that the improved amino acid and carbohydrate metabolism could provide adequate nutrients for the lactation activity of dairy cows. Further studies should aim to extract the specific composition and investigate the underlying mechanisms in the milk synthesis.

## CONCLUSION

Dandelion supplementation could improve lactation performance, and elevate the plasma carbohydrate and amino acids metabolism and antioxidative activity. Supplementation of 200 g/d dandelion is recommended for lactating dairy cows.

## Figures and Tables

**Table 1:** Effects of dietary dandelion supplements on feed intake and lactation performance in primiparous dairy cows

Item	Dandelion (g/d)				SEM	p-value^1)^		
	
	0	100	200	400		T	L	Q
DMI (kg/d)	20.6	20.7	20.8	20.6	-	-	-	-
Milk yield (kg/d)								
Raw	32.9^b^	33.9^ab^	34.7^a^	33.3^b^	0.26	0.03	0.63	0.04
ECM^2)^	32.6^b^	33.6^ab^	34.8^a^	33.2^b^	0.35	0.04	0.83	0.03
Fat	1.27	1.32	1.37	1.30	0.032	0.45	0.78	0.37
Protein	1.05	1.08	1.12	1.07	0.021	0.39	0.86	0.48
Lactose	1.66^b^	1.70^b^	1.75^a^	1.68^b^	0.034	0.05	0.75	0.05
Content (g/100 g)								
Fat	3.87	3.89	3.94	3.90	0.024	0.48	0.78	0.81
Protein	3.27	3.25	3.29	3.27	0.075	0.62	0.29	0.56
Lactose	5.14	5.12	5.15	5.12	0.047	0.49	0.87	0.50
MUN (mg/dL)	11.4	10.8	11.1	12.0	0.39	0.16	0.55	0.30
SCC (10^3^/mL)	199.2^a^	159.7^b^	147.3^b^	105.0^b^	0.10	<0.01	0.01	0.51

SEM, standard error of the mean; DMI, dry matter intake; MUN, milk urea nitrogen; SCC, somatic cell count.

1) T, treatment; L, linear; Q, quadratic.

2) ECM = 12.55×fat yield (kg/d)+7.39×protein yield (kg/d)+5.34×lactose yield (kg/d).

a,b Mean in a row with different superscripts are significantly different (p<0.05).

**Table 2:** Effects of dietary dandelion supplements on plasma variables in primiparous dairy cows

Item	Dandelion (g/d)				SEM	p-value^1)^		
	
	0	100	200	400		T	L	Q
Total protein (g/L)	73.9	73.5	74.8	72.2	0.59	0.54	0.79	0.37
Albumin (g/L)	34.4	34.5	33.7	34.0	0.27	0.70	0.71	0.47
Globulin (g/L)	39.4	39.0	41.0	38.2	0.76	0.63	0.86	0.34
Bilirubin (μmol/L)	3.60	3.78	3.76	3.96	0.082	0.49	0.89	0.48
Glucose (mmol/L)	3.45^b^	3.50^b^	3.60^a^	3.56^a^	0.023	0.22	0.83	0.05
Creatinine (μmol/L)	60.3	61.6	63.2	63.7	0.95	0.58	0.94	0.59
Triglyceride (mmol/L)	0.15	0.16	0.17	0.15	0.012	0.20	0.47	0.33
ALT (U/L)	29.0	28.6	26.5	28.3	0.54	0.39	0.86	0.57
AST (U/L)	87.6	89.4	96.3	87.3	1.88	0.96	0.29	0.48
ALP (U/L)	48.5	46.1	47.4	39.6	1.56	0.44	0.59	0.37
BUN (mmol/L)	4.88	4.74	5.31	4.96	0.123	0.28	0.47	0.39
NEFA (μmol/L)	137.2	155.6	145.3	155.0	5.34	0.59	0.86	0.39
MDA (μmol/L)	3.93^a^	2.95^b^	2.13^b^	2.04^b^	0.020	0.05	0.01	0.21
SOD (U/mL)	163.5^b^	165.1^b^	168.6^a^	169.9^a^	1.83	0.04	0.05	0.85
GSH-Px (U/L)	216.9^b^	228.4^b^	249.2^a^	222.8^b^	5.04	0.03	0.47	0.05
T-AOC (mmol/L)	0.52	0.53	0.52	0.52	0.011	0.93	0.93	0.58

SEM, standard error of the mean; ALT, alanine aminotransferase; AST, aspartate aminotransferase; ALP, alkaline phosphatase; BUN, blood urea nitrogen; NEFA, nonesterified fatty acid; MDA, malondialdehyde; SOD, superoxide dismutase; GSH-Px, glutathione peroxidase; T-AOC, total antioxidant capacity.

1) T, treatment; L, linear; Q, quadratic.

a,b Mean values within treatment with different superscripts are significantly different (p<0.05).

**Table 3:** Identification of significantly different metabolites in plasma between the CON^1)^ and DAN^1)^ groups

Metabolites	Similarity	VIP	FC^2)^	p-value
Dodecanol	253	1.75	2.63	<0.01
9-Fluorenone	385	1.52	2.05	0.01
Glucose	850	2.26	2.00	<0.01
Ribose	713	1.23	1.94	0.03
Valine	905	1.58	1.81	0.03
Salicylic acid	451	1.37	1.68	0.02
Thioctamide	257	1.60	1.63	0.04
Glutamic acid	690	1.14	1.54	0.02
Phenylalanine	836	1.06	1.47	0.03
2-Amino-3-phosphonopropanoic acid	292	2.15	1.34	0.01
Dihydrocarveol	313	2.13	1.31	0.01
Cytidine-monophosphate	466	1.57	1.26	0.04
1-Methylhydantoin	202	1.84	1.22	0.04
Palmitic acid	935	1.49	1.21	0.04
Glucose-1-phosphate	777	1.72	1.17	0.05
Mannose	888	1.60	1.16	0.03
2-Hydroxypyridine	887	1.79	1.15	0.03
Cystine	232	1.64	1.14	0.04
Methylmalonic acid	572	1.48	1.10	0.04
Maleamate	452	1.69	0.59	0.03
Digitoxose	249	1.87	0.52	0.05
Threonine	539	1.92	0.43	0.03
Indolelactate	533	1.82	0.39	0.03

VIP, variable importance in projection.

1) CON, control group; DAN, 200 g/d of dandelion supplemented group.

2) FC = fold change, mean value of peak area (DAN/CON).
