# Supplementary material for: Effects of dandelion (Taraxacum sp.,) supplements on lactation performance, antioxidative activity, and plasma metabolome in primiparous dairy cows
Source: Anim Biosci. 2022 Sep 2;36(2):229–37. doi: 10.5713/ab.22.0061 (PMC9834724; doi:10.5713/ab.22.0061)
Supplement: Supplementary file 1 [file ab-22-0061-suppl.pdf]

Supplemental Table 1. Ingredients and chemical composition of TMR.

Ingredient	% of DM	Chemical composition	% of DM
Corn silage	30.36	CP	16.68
Alfalfa hay	13.68	NDF	37.89
Corn grain	12.70	ADF	19.81
Oat hay	3.26	EE	2.92
Cotton seed	3.26	Ash	7.22
Beet pulp	5.54	NE_L[2], Mcal / kg of DM	1.68
Soybean meal	9.12		
DDGS[1]	4.33		
Flaked corn	9.77		
Rapeseed meal	2.93		
Extruded soybean	1.63		
Concentrate[3]	3.42		

[1] DDGS: distillers dried grains with solubles.

[2] Net energy for lactation, calculated based on the Ministry of Agriculture of China recommendations (MOA, 2004).

[3] Concentrate includes: 28.68% fat powder, 10.52% $Ca(HCO_3)_2$, 19.12% $NaHCO_3$, 9.56% stone powder, 11.47% salt, 1.53% rumen protected methionine, 4.78% yeast, 4.78% yeast culture, 4.78% MgO, 4.78% premix. Premix includes (per kilogram of DM): 17 KIU of Vitamin D, 73 KIU of Vitamin A, 1200 IU of Vitamin E, 60 mg of Co, 20 mg of Se, 40 mg of Fe, 255 mg of Cu, 708 mg of Mn, 40 mg of I.

Supplemental Table 2. The relative proportion of main bioactive compounds (% of total abundance of bioactive compounds) in dandelion (DAN) using LC/MS.

Bioactive Compounds	Proportion, %	Bioactive Compounds	Proportion, %
β-D-Glucopyranoside	12.32	Myristic acid	0.42

Caffeic acid	9.57	Tartaric acid	0.38
7,8-Dihydroxyflavone	8.61	Methylsuccinic acid	0.38
Luteolin-4’-*O*-glucoside	7.96	Methyl palmitoleate	0.27
Rutin	7.62	Heptadecanoic acid	0.16
9Z,11E-Linoleic acid	5.89	L-Valine	0.11
Choline	5.60	2-Hexenal	0.09
Proline	5.36	Palmitic acid	0.08
Trigonelline HCl	4.11	Piperidine	0.08
Malic acid	3.88	L-Phenylalanine	0.07
Valine	3.78	Sucrose	0.05
Trans-Vaccenic acid	3.32	L-Glutamic Acid	0.04
Phenylalanine	2.94	Chlorogenic acid	0.04
Methyl vanillate	2.65	Aspartate	0.04
Quercetin	2.58	Nicotinamide	0.04
Stachydrine	1.75	Dehydrocostus lactone	0.04
L-Isoleucine	1.63	Phthalic anhydride	0.04
9-Trans-Palmitelaidic acid	1.54	L-Carnitine	0.04
D-Gluconic acid	1.53	Isoimperatorin	0.03
Citrate	1.28	Tyrosine	0.03
Methyl Heptadecanoic acid	0.98	Formononetine	0.03
Mannitol	0.86	L-Theanine	0.02
Adenosine	0.64	Sarracenin	0.02
Nicotinic acid	0.55	p-Coumaraldehyde	0.01
L-Tryptophan	0.53	Decanoic acid	0.01

These tables were published in our previous study.

Li Y, Lv M, Wang J, Tian Z, Yu B, Wang B, Liu J, Liu H. Dandelion (*Taraxacum mongolicum* Hand.-Mazz.) Supplementation-Enhanced Rumen Fermentation through the Interaction between Ruminal Microbiome and Metabolome. Microorganisms. 2020 Dec 31;9(1):83. doi: 10.3390/microorganisms9010083. PMID: 33396441; PMCID: PMC7823719.
